# Building Healthy Start Grantees’ Capacity to Achieve Collective Impact: Lessons from the Field

**DOI:** 10.1007/s10995-017-2373-1

**Published:** 2017-11-24

**Authors:** Kimberly Bradley, Karuna S. Chibber, Naima Cozier, Peggy Vander Meulen, Colleen Ayres-Griffin

**Affiliations:** 1 Healthy Start EPIC Center, ZERO TO THREE, Washington, DC USA; 2 Healthy Start EPIC Center, JSI Research and Training Institute Inc., Boston, MA USA; 3 Healthy Start Grantee, Strong Beginnings, Grand Rapids, MI USA; 4 Healthy Start Grantee, Tulsa Healthy Start, Tulsa, OK USA

**Keywords:** Collective Impact, Capacity building, Community Action Network, Peer learning, Healthy Start

## Abstract

*Purpose* While Healthy Start has emphasized the need for multi-sectorial community engagement and collaboration since its inception, in 2014 Healthy Start adopted Collective Impact (CI) as a framework for reducing infant mortality. This paper describes the development of a peer-focused capacity-building strategy that introduced key elements of CI and preliminary findings of Healthy Start grantees’ progress with using CI as an approach to collaboration. *Description* The Collective Impact Peer Learning Networks (CI-PLNs) consisted of eight 90-min virtual monthly meetings and one face-to-face session that reviewed CI pre-conditions and conditions. Evaluation sources included: a facilitated group discussion at the final CI-PLN exploring grantee CI and CAN accomplishments (n = 57); routine evaluations (n = 144 pre, 46 interim, and 40 post PLN) examining changes in knowledge and practices regarding CI; and post CI-PLN implementation, three in-depth interviews with grantees who volunteered to discuss their experience with CI and participation in the CI-PLN. *Assessment* CI-PLN participants reported increased knowledge and confidence in the application of CI. Several participants reported that the CI-PLN created a space for engaging in peer sharing challenges, successes, and best practices. Participants also reported a desire to continue implementing CI and furthering their learning. *Conclusion* The CI-PLNs met the initial goal of increasing Healthy Start grantees’ understanding of CI and determining the initial focus of their efforts. By year five, the EPIC Center anticipates Healthy Start CANs will have a sustainable infrastructure in place that supports the established common agenda, shared measures, and ongoing and meaningful inclusion of community members.

## Significance

Collective Impact is an approach to collaboration that aims to address complex social problems. Many communities, including Healthy Start programs, have engaged in community engagement and collaboration for years. The CI framework enhances traditional collaboration practices to encourage a culture of shared leadership, deeper community engagement, increased accountability, system-wide shared vision, and support to facilitate and coordinate the process. This paper contributes to lessons learned with regard to distance learning and effective virtual technical assistance delivery methods for national audiences that are at varying levels of understanding and stages of implementation.

## Purpose

From its inception in 1991, the Healthy Start projects recognized the importance of community engagement and collaboration in addressing infant mortality and birth outcome disparities. In 1994, the National Center for Education in Maternal and Child Health published a Healthy Start Initiative series of informational booklets. The first booklet was devoted to Consortia Development. “The key reason that collaboration is essential to combat infant mortality is found in the complexity of the problem itself. Because infant mortality is affected by socioeconomic conditions such as poverty, inadequate housing, unemployment, racism, and violence, no single organization can solve the problem. For many pregnant women, personal issues such as substance abuse, youth, and a general feeling of hopelessness compound these difficulties. Only a collaborative effort within the community can create the long-term vision needed to attack the problem from a myriad of angles. This is not a simple task.” (McCoy-Thompson 1994)

While Healthy Start has encouraged multi-sectorial collaboration since its inception, in 2014 it was decided that changing the name of these collaborative bodies from the consortia to “Community Action Network” (CAN), would better described the commitment to action. The 100 Healthy Start CANs across the country are diverse in composition, strength of partnerships, area of focus, and leadership. There is significant variation in the history of the Healthy Start consortiums/CANs; some have been in existence for over 20 years and others being established for the first time. In 2014, Healthy Start adopted Collective Impact (CI) as a more structured framework for large-scale change, such as that required for reducing infant mortality. Healthy Start CANs now serve as the vehicle for achieving Collective Impact.

While many United States communities have adopted CI to solve complex problems, this is the first time within the US that CI has been used by a federally funded program at this scale. This paper describes a capacity-building approach and preliminary findings from the application of this approach with Healthy Start sites.

## Description

### The Theory of Change in Applying a Collective Impact Approach

Collective Impact is defined as, “A framework to tackle deeply entrenched and complex social problems. It is an innovative and structured approach to making collaboration work across government, business, philanthropy, non-profit organizations and citizens to achieve significant and lasting social change” (Collaboration for Impact 2015). The founders emphasize the importance of community coalition efforts as groundwork for CI. CI efforts are most effective when “they build from what already exists; honoring current efforts and engaging established organizations, rather than creating an entirely new solution from scratch” (Hanleybrown et al. 2012). Healthy Start’s long history of collaborating through consortiums set a strong foundation for CI.

Collective Impact includes three pre-conditions: influential champions, adequate resources, and urgency of issue, and five conditions: common agenda, shared measurement, mutually reinforcing activities, continuous communication, and backbone support (Kania and Kramer 2011).

In 2012, this framework was further expanded to include four phases and four components for success: governance and infrastructure, strategic planning, community involvement, and evaluation and improvement. Though CI appears to be a linear process, experienced users emphasize that system level change is complex and dynamic and takes times (Hanleybrown et al. 2012).

### Healthy Start and Collective Impact

**Fig. 1:**
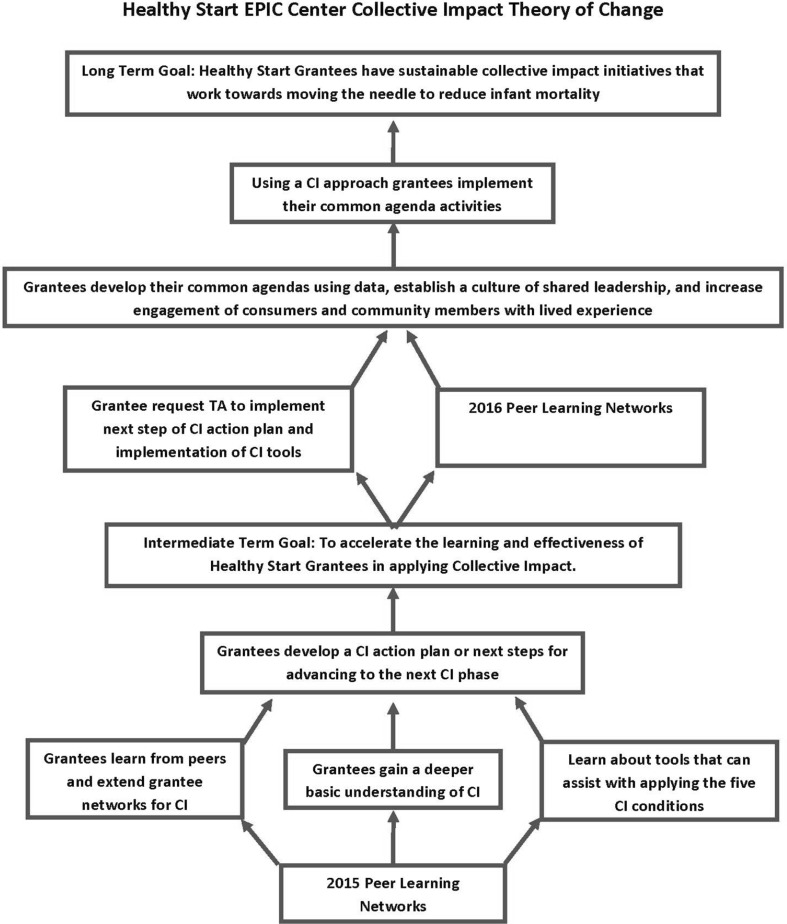
Healthy Start EPIC Center’s Collective Impact theory of change

The Healthy Start EPIC Center (EPIC Center) serves as Healthy Start grantees’ capacity-building provider which includes technical assistance for collective impact. After extensive consultation with Tamarack, an organization internationally recognized for their expertise in CI, it was decided to launch a peer learning series that provided a forum for grantees to learn the core principles of CI and share implementation experiences. Using a theory of change model, a peer learning series called the Collective Impact Peer Learning Networks (CI-PLNs) was developed. The CI-PLNs provided a dedicated forum for grantees to share their experiences applying CI within the context of their community. The goals of the PLN series were threefold:

1. Develop a deeper understanding of CI;
2. Learn about CI tools;
3. Develop a CI action plan (Fig. 1).

After seeking feedback from Healthy Start grantees and the federal Healthy Start Division staff, six CI-PLNs were established stratified according to grantees’ CAN application of CI and the delineation of geographical areas (urban, rural, and border):

1. Applying CI to an established CAN in a rural area
2. Healthy Start projects joining existing CAN/CI efforts
3. New Healthy Start projects or CAN
4. Applying CI to a CAN in a border community
5. Applying CI to an established CAN in Urban Area (there were two groups in this area divided by time zones, then federal regions)

**Fig. 2:**
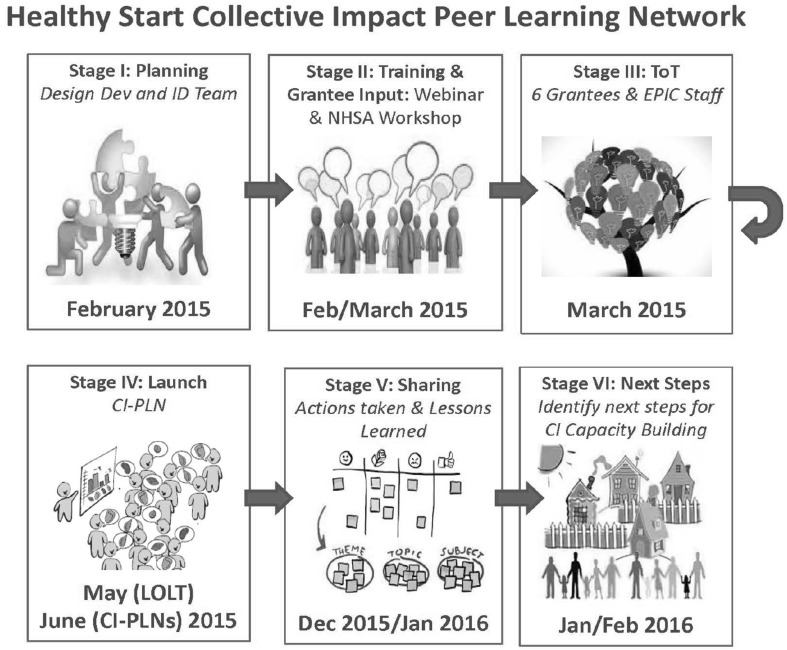
Phases and timeline of the CI-PLN planning and implementation process

A few key features of the CI-PLN structure were:

- Each CI-PLN was co-facilitated by a grantee and an EPIC center staff member/consultant. This pairing balanced the context and content experience.
- Prior to launching the CI-PLN, co-facilitators received customized training with an accompanying Implementation Tool Kit developed by Tamarack. The training focused on the core elements of establishing a group culture that facilitated relationship building, trust and sharing.
- All the co-facilitators met monthly as a full team and in pairs to plan and debrief CI-PLN meetings, develop new resources, and make course corrections as needed (Fig. 2).

The CI-PLNs began in June 2015 and met virtually using a web meeting platform. To introduce the series, the EPIC Center sponsored a webinar titled “Launching Our Learning Together” that included the co-facilitators and Tamarack representatives, Healthy Start Division staff, and National Healthy Start Association staff. A total of 145 individuals representing 80 grantees registered to participate in the series. Even after initial registration closed the groups remained “open” throughout the series to encourage participation and accommodate staff turnover and scheduling conflicts.

**Fig. 3:**
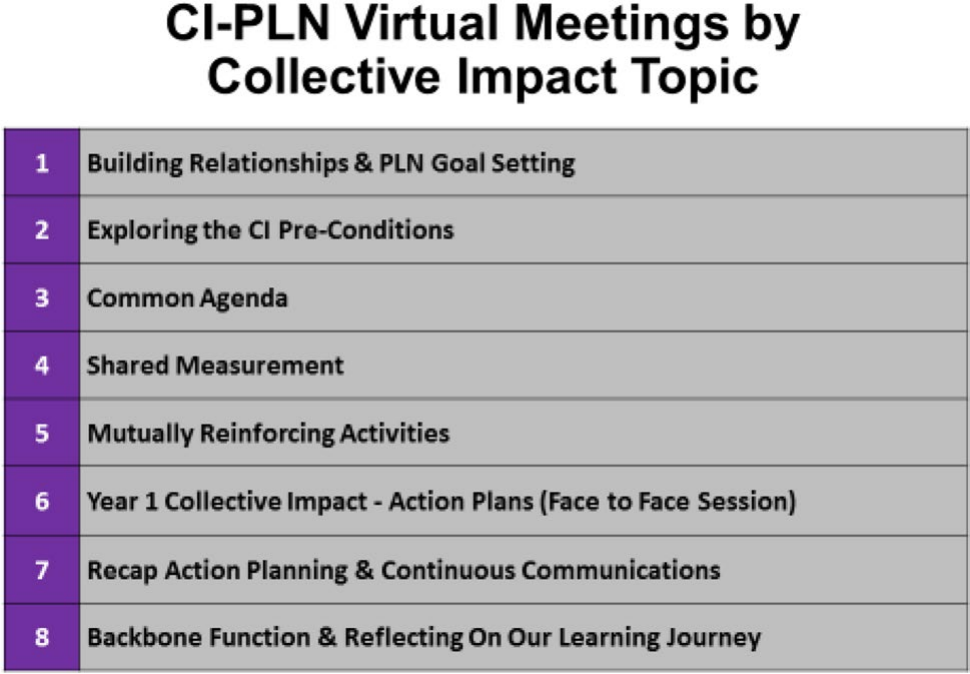
CI-PLN monthly meeting topics and focus areas

A series of seven 90-min virtual meetings occurred monthly. Grantees worked through the CI pre-conditions and conditions and had one in-person session on action planning which was held during the Healthy Start Convention in November 2015 (Fig. 3).

Between meetings, grantees were encouraged to apply CI tools (Available on the EPIC Center Website, http://healthystartepic.org/hear-from-your-peers/collective-impact-pln/) related to the CI condition of focus during that month. This provided a basis for discussion and exchange on the next CI-PLN call. By the end of the series in January 2016, the goal was that each grantee would have had an opportunity to develop a CI Action Plan using the tool provided.

## Assessment

### Methods

Data were collected from Healthy Start grantees and CI-PLN co-facilitators using various data collection methods including: participation tracking data, a facilitated group discussion exploring grantee perspectives on accomplishments (n = 57); three in-depth interviews with grantees who volunteered to answer additional questions about their experiences; evaluation surveys (n = 141 pre, 46 interim, and 40 post PLN) examining changes in knowledge and practices regarding CI; and co-facilitator assessments of the PLN process (n = 9). All data were self-reported and the survey response rate declined over time (pre-registration survey = 83%, midterm = 27%, final survey = 24%). This study was reviewed and approved by John Snow, Inc.’s Institutional Review Board and received exempt status since it does not meet the definitions of human subjects research.

### Findings

Findings provide insights regarding the application of a PLN to support CI implementation.

### Participation

Participation was voluntary, tracked monthly and expected to fluctuate after the first call since the series was voluntary and grantees could decide whether or not to participate and which PLN group best met their needs. Initially, 145 individuals representing 80 grantees signed up for the PLN series, however 150 individuals representing 85 grantees were consistent participants. Participation was defined as attending at least two events. Individuals participating in only one event were not included in participant counts. Table 1 shows the distribution of participation by meetings aggregated across PLNs. For the cohort of individuals who attended at least two CI-PLN events (monthly calls and in-person session), on average, those participants attended half of all events (4.2 of the 8 events). Though participation declined over time, even at the last PLN, 50% of all Healthy Start grantees were represented.

**Table 1:** Distribution of participation across PLN series

Summary statistics (All PLNs)	Total registered (participating in at least two events)	Call #1 attended	Call #2 attended	Call #3 attended	Call #4 attended	Call #5 attended	In person #6 attended	Call #7 attended	Call #8 attended
% unique grantees who participated	85	76%	75%	73%	68%	66%	68%	64%	54%
% of individuals who participated	150	71%	69%	59%	51%	52%	52%	45%	38%

### CI-PLN Participation Increased Some Grantees’ Knowledge and Confidence About CI

Grantees reported that participating in the PLN increased their knowledge about CI, tools, and their confidence in explaining CI to CAN members. Most grantees also said that they better understood the purpose of CANs, and how to implement a CI approach. About a third reported being able to better focus their strategies, identify gaps in their CI/CAN development efforts, and plan next steps. Expressing these sentiments one participant said:

I appreciate the PLN. We have been able to formalize what we’re doing and focus on the purpose that we are coming together for and get things accomplished.

Another highlighted a specific tool that had been very useful to her organization:

The Stakeholder Engagement Tool helped us facilitate conversations with CAN members about the various ways and levels they wanted to be involved.

Elaborating on the strengths of the CI approach, interview respondents said that adopting CI brought greater recognition of each partner’s value and expertise, and how collaboration would yield better results. One respondent highlighted how CI enabled a more equal and “bottom-up” planning process and provided a structure for continuous communication.

**Fig. 4:**
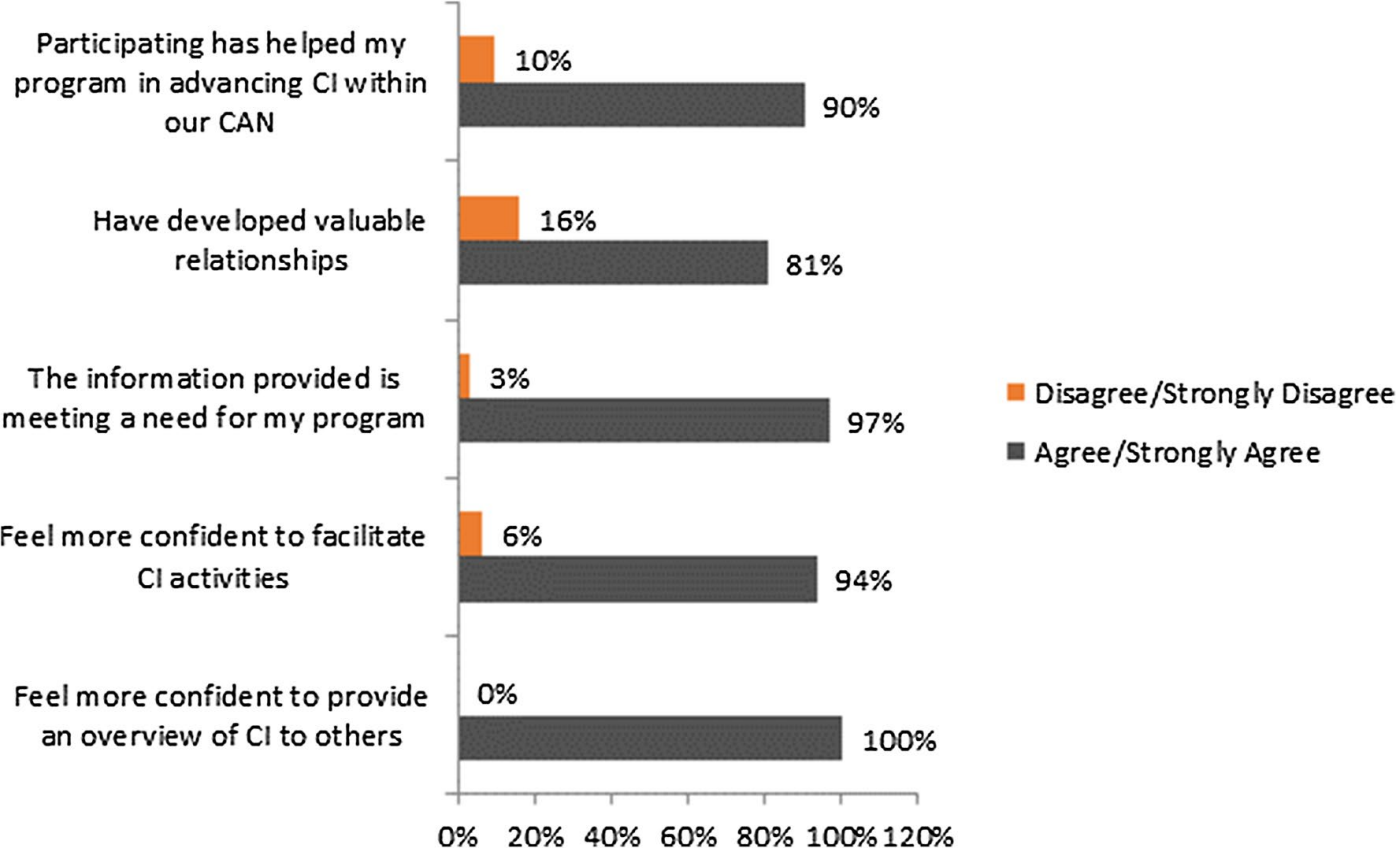
Usefulness of PLN: perspective of respondents completing final evaluation survey

Analysis of evaluation surveys revealed similar findings. Respondents reported greater confidence in their ability to provide an overview of CI (88% interim −100% post PLN), and greater confidence in facilitating CI activities with CANs (65% interim −94% post PLN) (data not shown). Additionally, respondents who completed the final survey reported an increase in confidence and overall usefulness of the information provided through the PLNs (Fig. 4). Most respondents reported that the CI-PLN was enhancing their program’s ability to advance CI with their CANs (90%), and 94% reported being more confident in facilitating CI-related activities.

**Fig. 5:**
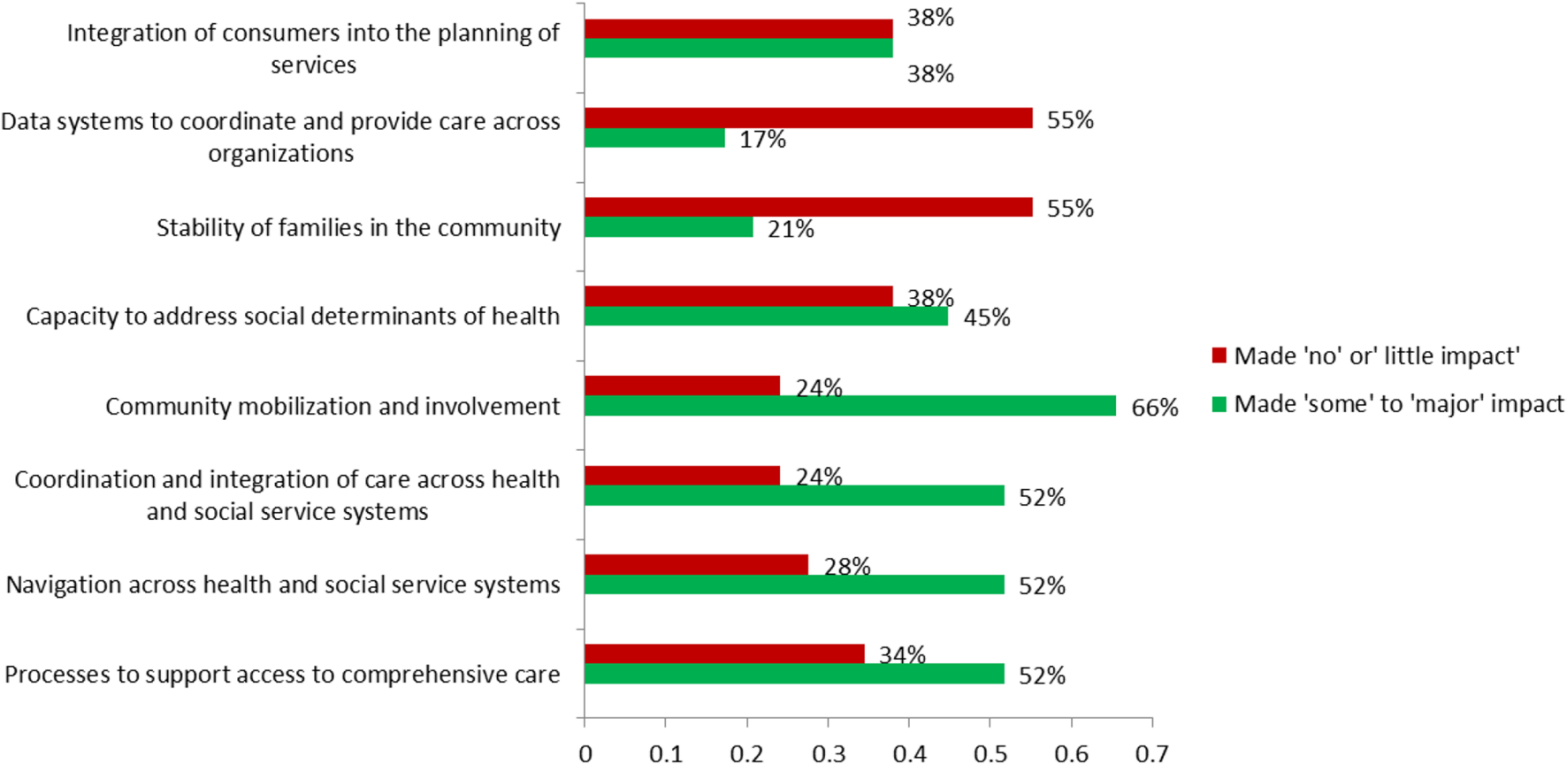
Changes in CAN operations: perspective of respondents completing final evaluation survey

The data also suggest that for some grantees, participating in the PLN enabled their CAN to make some impact over the past year. Figure 5 shows areas of impact. Not surprisingly, these related to the building blocks of CAN efforts, such as community mobilization, coordination across services and establishment of systems. favorably impacted their community.

### Participating in the CI-PLN Resulted in Concrete Accomplishments for Several Grantees

Most participants who provided feedback reported progress. For grantees earlier on in their efforts, accomplishments ranged from creating momentum and spreading awareness about CI to holding a CAN meeting to “finally getting buy-in from CAN members to create a common agenda...” Two key informants said their biggest accomplishments were building trust with CAN partners, ensuring that partners were invested, and engaging partners from new sectors, including increasing consumer participation.

For participants further along in their CAN/CI efforts, accomplishments included developing CI action plans and working groups. A few reported progress on their shared measurement and evaluation by “creating an integrated system around benchmarks and requirements.”

### CI-PLN Participation Facilitated Sharing, Learning, and Relationship Building

Participants reported that the CI-PLN created a space for peer sharing and learning. Participants emphasized the importance of hearing from their peers, explaining that it affirmed their work and provided insight into novel approaches that could be adapted to their context. One participant said:

Hearing from other groups about their progress in their CI efforts, what tools they have used to broaden participant representation for example, has given us ideas for what we should do/not do going forward. (After the in-person sessions) I have a direct contact that I can reach out to for guidance.

### Participants Expressed a Strong Desire to Continue Implementing CI

More than two-thirds of the grantees participating in the interviews and group discussion reported a strong desire to continue implementing CI. Participants described a range of next steps, including meeting with CANs to share CI tools; establishing a fully operationalized CAN; finalizing a common agenda; expanding recruitment of CAN members; increasing diversity and consumer participation; and improving shared measurement systems.

### Best Practices and Areas of Improvement for Subsequent PLNs/CI Implementation

Co-facilitators described various best practices. For example, co-facilitators felt the facilitation structure of an EPIC Center staff and Healthy Start grantee was a success. It “helped to establish trust and lent credibility,” and ensured that information-sharing and discussions were grounded in grantees’ experiences. Additionally, more than half of the co-facilitators reported that technology had been critical in facilitating discussion. Other best practices included initiating the CI-PLN with in-person training for co-facilitators, scheduling time for co-facilitators to share emerging findings and jointly plan sessions, and allowing time for participant discussion and sharing at each session.

Co-facilitators said that an important lesson learned was the need for additional in-person opportunities to build and strengthen relationships among grantees, particularly to increase trust and sharing. They also suggested improving planning by realistically outlining the time commitment and level of effort. For example, none of the co-facilitator teams had time to use the additional consultation made available by Tamarack Institute. Similarly, the participation requirement for grantees to develop an action plan was later dropped. Future PLNs should ensure that participation requirements are better aligned with grantee skill, level of effort, and curriculum development. Another common theme was the need for better stratification of grantees (e.g., based on grantees’ stage of CI/CAN efforts). Co-facilitators said that working with a smaller and more homogenous group would have enabled them to better understand each grantee and their respective contexts, allowing them to offer a more tailored training. As future CI-PLNs are planned and developed this feedback will be taken into consideration.

## Limitations

These findings should be viewed in light of several limitations. First, CI-PLN participation was not consistent and declined over time. Second, the evaluation response rate for the midterm and final surveys were low and, as such, results are not representative of all CI-PLN members. Third, findings suffer from the limitations of self-reported data. Fourth, findings are from the perspective of an early implementation of CI and therefore do not provide insights on the effectiveness of CI in achieving Healthy Start objectives. Fifth, it is widely established that intention is different from actual practice. Though grantees reported changes in practices, it remains to be seen if lessons learned fully translate into practice. Finally, the analysis contained herein includes only two perspectives, grantees and co-facilitators. Additional research is needed to understand the perspective of other key stakeholders, including federal staff. These limitations aside, the findings do provide insights into the feasibility of PLNs as a strategy for capacity building and dissemination of best practices. All the evaluation efforts described herein were designed to enable real-time learning and not for rigorous evaluation. Further research is needed to establish the association between PLN participation and impact; for example, a PLN designed and implemented for a pre-determined time period, with a fixed group of participants will provide much needed information regarding the ‘dose effect’ of PLN participation on the desired outcomes. Additional research may also be needed to better understand CI implementation effects at the individual grantee level and nation-wide, and whether the changes are sustained over time.

## Conclusions

Fully applying CI is a lengthy process. The EPIC Center’s short term goal was that grantees gain a deeper understanding of CI and decide which pre-condition, condition, or phase on which to focus their initial efforts. Based on the findings, the CI-PLNs may have been successful in assisting grantees with this initial goal. The findings also offer insights regarding several best practices for using PLNs as a capacity building strategy. By year five, the EPIC Center anticipates Heathy Start CANs who have ensured the CI three pre-conditions and five conditions will have a sustainable collaborative infrastructure in place to reduce infant mortality rates.
